# Event and Comment

**Published:** 1915-08

**Authors:** 


### DENTAL REGISTER

Vol. LXIX AUGUST, 1915 No. 8

### EVENT AND COMMENT

AMERICAN DENTAL HOSPITAL. Don't fail to read the letter from Dr. Le Cron in this issue in regard to the work American dentists are doing for wounded soldiers in Paris, and don't fail to make a contribution to help carry on this good work.

EASTMAN DENTAL BENEFACTION. In another place in this issue we print an account of another large gift to suffering humanity in the form of a dental infirmary for the treatment of dental diseases of children's teeth, and a training school for the prevention of oral deformations. The gift is a building ample in proportions and equipment for the care of the teeth of poor children in the city of Rochester, N. Y., and an endowment sufficient to carry on this work in the most effective manner. The donor is Mr. George Eastman, the man who made the Kodak famous. He certainly has given a lot of people much pleasure in popularizing amateur photography, and now he is planning to make untold numbers happier and more useful citizens by this great philanthropy. We dentists who know of the immense advantage a child will have in the battle for life who has a perfect set of teeth, can readily put a proper valuation on this benevolent action, but it may not be so apparent to those who do not come in contact with the tremendous loss of efficiency in this world caused by diseased dental organs, especially in children. Personally we have come to the conclusion, after a long study of this problem, that the superman can not materialize until due attention has been given to the normal development of the oral and respiratory organs. A competent dentist co-operating with a similarly qualified nose and throat physician and a school teacher who knows some other things besides the "three Rs" will do more to bring the perfect physically developed youth than almost any other combination that we can think of. Of course we take it for granted that the child has at least the average well regulated home life. We suppose the Rochester Dental Society, the pioneers in Oral Hygiene work in the public schools, are responsible in large measure for this great benefaction and the whole dental profession offers its cordial congratulations. We should all take courage and believe that what has happened in Boston and Rochester may materialize in New York, Chicago and hundreds of other likely places. The Lord be praised for men of means who are far-sighted enough to use their talents where they will bring the most profitable returns in human happiness. If the people of Europe had taken the precaution to have had their rulers' teeth properly taken care of when they were young they would be today a race of humanitarians instead of brutal murderers—perhaps. With good digestions they would have been at least less quarrelsome. The profession will be interested to see how this new enterprise will be handled; but we feel sure that the Rochester men who have been living with this problem for the last ten years have gained some experience that will enable them to see the way clearly to the best forms of administration. Their experience and the wisdom of the man who has made the work possible can be depended upon to devise the most practicable scheme for successfully carrying out this splendid idea. We all are deeply hopeful for a successful outcome.

